# Service guidelines, models, and protocols for integrating rehabilitation services in primary healthcare in Brazil, Russia, India, China, and South Africa: a scoping review

**DOI:** 10.1080/09638288.2023.2290210

**Published:** 2023-12-09

**Authors:** Lebogang Maseko, Hellen Myezwa, Natalie Benjamin-Damons, Denise Franzsen, Fasloen Adams

**Affiliations:** a Occupational Therapy Department, Faculty of Health Sciences, School of Therapeutic Sciences, University of the Witwatersrand, Gauteng, South Africa; b Physiotherapy Department, Faculty of Health Sciences, School of Therapeutic Sciences, University of the Witwatersrand, Gauteng, South Africa; c Division of Occupational Therapy, Department of Health and Rehabilitation Sciences, Faculty of Medicine and Health Sciences, Stellenbosch University, Stellenbosch, South Africa

**Keywords:** Integrated services, people-centred health systems, primary healthcare, rehabilitation, universal health coverage, occupational therapy, physiotherapy, speech therapy and audiology

## Abstract

**Purpose:**

The WHO emphasises that rehabilitation services must be integrated into primary healthcare as an inherent part of universal health coverage. However, there is limited research on the integration of rehabilitation services in primary healthcare in low- and middle-income countries. The purpose of this paper is to identify and describe the literature on service guidelines, models, and protocols that support the integration of rehabilitation services in primary healthcare in the BRICS countries (Brazil, Russia, India, China, and South Africa).

**Methods:**

A scoping review guided by Arksey and O’Malley’s framework was conducted. Structured database and website searches identified published and unpublished records from 2010, which were subjected to eligibility criteria. Mendeley, JBI SUMARI, and Microsoft Excel were used to extract and synthesise the data.

**Results:**

The search strategy identified 542 records. Thirty-two records met the inclusion criteria. Shared care and community-based rehabilitation were the most reported practice models, and the implementation of the models, guidelines, and protocols was mostly described in mental health services.

**Conclusion:**

This review discusses BRICS countries’ rehabilitation service guidelines, models, and protocols for primary healthcare integration and implementation challenges. Rehabilitation professionals should rethink, realign, and apply existing models because of the lack of primary healthcare integration directives.

## IMPLICATIONS FOR REHABILITATION

- The integration of rehabilitation services in low-resourced and remote settings can be improved by involving community health workers and community rehabilitation workers in transdisciplinary teams.
- Peer support workers and community health workers can improve rehabilitation outcomes, particularly through shared care models that emphasize peer-to-peer learning, mentoring, and coaching.
- Self-management interventions can have a positive impact on functional outcomes.
- Integrated rehabilitation services in primary healthcare can be supported through community-based rehabilitation, which emphasises community involvement and engagement.

## Introduction

Changing global health trends over the last decade are placing greater demands on health and social systems, which supports the need for increased rehabilitation services for a variety of health conditions for 2.41 billion people globally [1]. Individuals with chronic diseases recognise that one of the biggest flaws of current health systems is the absence of integrated rehabilitation services at the primary healthcare (PHC) level which results in negative health outcomes [2,3]. Integrating rehabilitation into the health system at the PHC level is critical to bridge the gap in service delivery, particularly in low- and middle-income countries where health systems usually prioritise curative medical care [1].

However, despite the documented advantages of delivering rehabilitation services at PHC level, there is a dearth of evidence on how and to what extent rehabilitation services are integrated into PHC. This could be because the WHO Rehabilitation 2030 guidelines only recently highlighted rehabilitation services in PHC systems research [4]. In accordance with universal health coverage in several nations and the increased global emphasis on disability [5–7], these guidelines suggest rehabilitation services should be strengthened and integrated into PHC in accordance with current epidemiological trends and demographic shifts [4].

It is being argued that rehabilitation is a fluid and complex construct that requires varied levels of interdisciplinary collaboration to respond to changing socio-political discourse that influences the lives of people with disabilities [7]. The WHO operational framework for PHC provides indicators that can be used to address the integration of rehabilitation in PHC [8]. The integration of these services can be viewed from a variety of service delivery process perspectives, including practice models, service protocols, and guidelines [9–11]. To enhance the integration of rehabilitation in primary healthcare (PHC), it is important to thoroughly review and re-evaluate the current models, protocols, and guidelines. The process should consider various perspectives that carefully reflect on the specific context and the population served. Challenges that affect the integration of rehabilitation services in PHC have been identified and can be analysed in the context of the rehabilitation and health system building blocks, which include governance and leadership, finance, health workforce, technology, and information systems [12].

The literature identifies obstacles that hinder the capacity of health systems to provide rehabilitation services and address patients’ rights to access rehabilitation in PHC. Included in this are international and local policies that fail to address the extent to which rehabilitation services must be organised and delivered for integration to be realised [13–15]. The lack of direction to operationalise rehabilitation services within a PHC package [16–18] is further hampered by a shortage of a rehabilitation healthcare workforce [19–22] and varying approaches to service delivery [17,23,24]. Furthermore, medical health professionals’ perceptions and lack of understanding of disability, the PHC model, and a biomedical rather than biopsychosocial approach to healthcare are recognised as affecting rehabilitation services [24,25]. Limited research that specifically addresses the heterogeneity in the scope and the extent of interdisciplinary rehabilitation services, including the rehabilitation professions, namely occupational therapy, physiotherapy, and speech and language therapy (SLT), is another barrier to integrating rehabilitation services.

The limited prior research on the integration of rehabilitation services into PHC in low- and middle-income countries is based on a range of perspectives, including health system performance, service quality intervention, and health programmes [26–31]. However, there has been no systematic mapping of models, service guidelines, and protocols for integrating rehabilitation into PHC in low- and middle-income countries. Therefore, this scoping review aimed to identify and describe available models, service guidelines, and protocols for integrating rehabilitation services into PHC in Brazil, Russia, India, China, and South Africa (BRICS).

All five countries in the BRICS bloc are low- to middle-income countries with emerging economies and are individually implementing healthcare reforms to achieve universal health coverage [32]. The BRICS countries have significant pockets of poverty, marked discrepancies between income levels within their populations, and varying use of public and private health services [15]. Health challenges in these countries include an increase in the prevalence of non-communicable chronic diseases and high inequalities in health and healthcare access. In all these countries the public healthcare systems use community-based health workers with limited training to provide PHC services [33,34]. A comparative table of overall health policy, inception of PHC, rehabilitation integration, and rehabilitation professional training is presented in Table 1.

**Table 1.** A comparison of overall health policy, PHC inception, rehabilitation integration, and professional training in the BRICS countries.

Country	Overall health policy	Inception of PHC	Rehabilitation integration	Rehabilitation professional training
Brazil	Universal, free healthcare guaranteed by the 1988 Federal Constitution Primary care is one of the main pillars [1]	Family Health Strategy introduced in 1994 Community-based interdisciplinary teams provide PHC with a move away from clinics [1]	Well-advanced integration of rehabilitation in PHC with multidisciplinary teams including OT, PT, and SLT [2]	Started in the 1950s and 1960s Postgraduate courses introduced in 2010 [3]
China	Rehabilitation services included as part of health reforms since 2010 [4]	Wide network of PHC clinics and centres developed in the last 12 years [5]	Rehabilitation services mostly provided at hospitals or clinics [5]	Started in mainland China in 2010 PHC rehabilitation provided by midlevel workers trained in traditional Chinese medicine [6]
India	National Policy for Persons with Disability 2005 [7]	Public-private healthcare system [8]	Rehabilitation services still predominantly hospital-based [9]	Started in the 1950s Postgraduate courses introduced in 2000s [8]
Russia	Revenues generated from state and regional budgets, compulsory health insurance, and numerous enterprises	Federal Law on the Fundamentals of Health Protection governs PHC and rehabilitation	Rehabilitation services remain disjointed and need to be integrated into PHC [10]	Rehabilitation medicine has existed since 1917 the training of PTs OTs and SLTs started more recently [11]
SA	Framework and Strategy for Disability and Rehabilitation in South Africa 2015–2020 emphasizes need for integrated healthcare access [12]	Health services remain essentially clinic-based, curative, and siloed [13]	Rehabilitation services still predominantly hospital-based [13]	Started in the 1940s with postgraduate courses introduced in the 1970s [14]

Each country interprets the national and international rehabilitation guidelines differently and follows varying approaches to integrate rehabilitation into PHC, depending on their available resources and health infrastructure [35]. The BRICS countries have distinct policy directives to guide the integration of rehabilitation services, but implementation in some remains problematic [33].

Rehabilitation infrastructure, resources, services, and personnel vary across BRICS countries. Occupational therapy, physiotherapy, and SLT training are available in all five BRICS nations. Rehabilitation training has been offered for many years and is well established in South Africa, Brazil, and India, but in China and Russia, rehabilitation programmes are underdeveloped, in their infancy, and still expanding because they were only established during the last 10 years. The lack of clear integration guidelines and the variability in rehabilitation services highlight the need to identify and describe the literature on service guidelines, models, and protocols that support the integration of rehabilitation services in primary healthcare in the BRICS countries.

### Purpose of the scoping review

Arksey and O’Malley’s [36] and Levac et al.’s [37,38] scoping review methodology was used. Reporting was conducted according to the Preferred Reporting Items for Systematic reviews and Meta-Analyses extension for Scoping Reviews (PRISMA-ScR) [39]. The methodology consists of the five stages discussed in the following sections.

#### Stage 1: identifying the research question

The following research question guided the review: What are the existing service guidelines, practice models, and service protocols for the integration of rehabilitation services in PHC in the BRICS countries? The population, concept, and context (PCC) acronym [38] were used to define the research for this study.

#### Stage 2: identifying relevant studies

The search strategy was developed using an initial limited search of Medline and CINAHL. The text words and index terms from the results were used to create a comprehensive search strategy. Further relevant studies were identified through searches of various databases, including Wiley Online Library, Scopus, and ProQuest. Guidelines, protocols, policy reports, and newsletters were sourced from the WHO website and institutional websites. Additional articles were found through reference searches and database alerts. Lastly, LILACS was searched for BRICS papers using specific search terms (Table 2).

**Table 2.** Search terms.

Concept 1		Concept 2		Concept 3		Concept 4
“Rehabilitation”	AND	“Brazil”	AND	“Guidelines”	AND	“Primary health care”
OR		OR		OR		OR
“Occupational therapy”		“India”		“Clinical guidelines”		“Primary care”
OR		OR				OR
“Physiotherapy”		“China”		“Practice guidelines”		“Primary level of care”
OR		OR		OR		
“Speech and language therapy”		“Russia”		“Models”		
OR		OR		OR		
“Audiology”		“South Africa”		“Frameworks”		
OR		OR		OR		
“Community rehabilitation”		“BRICS”		“Protocols”		
OR						
“Physical therapy”						

The search strategy aimed to locate both published and unpublished studies. Filters were applied for database searches and records were included according to the eligibility criteria (Table 3). The search was conducted in September 2022 and updated in October 2023. The updated search yielded 22 additional studies, but none qualified for inclusion in the review.

**Table 3.** Eligibility criteria for selection of evidence/studies.

Eligibility criteria	inclusion	exclusion
Population (P)	Rehabilitation services, defined as Occupational Therapy (OT), Physiotherapy (PT), Speech Therapy (ST), and Audiology (Audio)	Any other rehabilitation services, such as podiatry, biokinetics, and others
Concept (C)	Service delivery guidelines or models, or protocols used at PHC level and rehabilitation, or services specifically designed for people with chronic conditions/disabilities.	
Context (C)	Brazil, Russia, India, China, South Africa	Other low- and middle-income countries
	Published/produced from January 2010	
	Primary Healthcare services	Hospitals, in-patient facilities, acute and sub-acute facilities
Publication status	Published peer-reviewed studies, and grey literature	
	Existing policies on disability and rehabilitation	
Language	English or translated into English	
Study designs	Literature looking at primary and secondary data as well as guidelines	

#### Stage 3: study selection

All identified citations were collated and uploaded into the Mendeley reference manager and duplicates were removed. Two independent reviewers screened the titles and abstracts against the inclusion criteria for the review. Potentially relevant studies were retrieved in full and their citation details were imported into JBI SUMARI [40]. Thereafter, the full text of selected citations was assessed in detail against the inclusion criteria. Any uncertainty that arose was resolved through discussion or with a third reviewer. Records were included in the study when there was agreement as to their suitability. The search results are presented in a PRISMA flow diagram [41].

#### Stage 4: charting the data

The researchers created a Microsoft Excel custom spreadsheet for data extraction, which was separately piloted by three reviewers and amended based on their suggestions. The final spreadsheet contained eight main headings, namely reference, country, aim of the service, methods, service providers/setting/context, participants, services described, and key findings from the source. The included studies were classified according to the National Health and Medical Research Council hierarchy of evidence [42], which grades studies from Level I (highest level) to IV (lowest level). The studies were not critically appraised as this is not typically done for scoping reviews [36,43].

#### Stage 5: extracting and reporting the results

Data were extracted based on three categories of interest, namely practice models [9], service protocols [11], and service guidelines [10] (Table 4). The authors of these studies presented the most detailed description and characterisation of the concepts relevant to rehabilitation services, which were used to guide an understanding of rehabilitation integration in PHC. The data extraction included descriptions and evidence for the integration of rehabilitation services in PHC as well as the challenges to the integration of these services. Records within each category were described based on the country of origin and the manner in which the concepts were applied in the integration of rehabilitation services.

**Table 4.** Description of concepts mapped.

Practice models [9]	The conceptual framework and philosophy that underpin healthcare delivery. Practice models can be regarded of as a relationship between client populations’ problems, the goals of healthcare practitioners, and the goals of healthcare systems [16].
Clinic	Physicians, nurses and rehabilitation professionals are co-located, resulting in a geographically defined team.
Outreach	Emanate from an institutional base and concentrate on providing professional services to people who could not access them in their usual institutional location.
Case management	A case manager based on a referral and intake assessment, marshals and coordinates the necessary services, including rehabilitation services, either in the patient’s home or other community location.
Shared care	Integration model where two providers with the same professional background (usually medical), one a specialist and one a generalist pair up in the provision of care, e.g., a psychiatrist and a general practitioner.
Self-management	Systematic provision of education and support by health care staff to increase service users’ skill and confidence in managing their health. Emphasises the patient as consumer and coordinator of his/her own services.
Community-based rehabilitation (CBR)	Based on a community development philosophy, where the role of rehabilitation professionals is to advocate for the issues of people with chronic diseases and disabilities and to assist in the mobilization of community resources and supports.
Service protocols [17]	Act as an impetus for quality teams to draw together health worker professional standards within a team: to improve communications; to improve the quality of service; to meet organisational goals.
Service guidelines [18]	Refer to any aspect of rehabilitation intervention which must be carried out in a certain manner to: adhere to organizational policies, adhere to professional standards, guarantee reliability of the rehabilitation process.

## Results

### Search results

**Figure 1.**
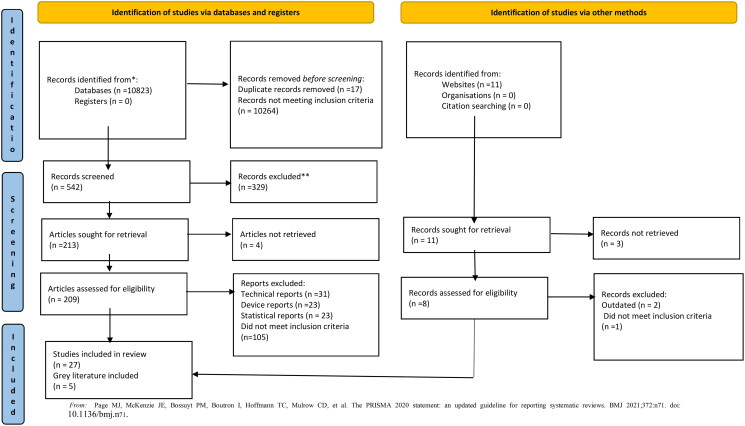
PRISMA 2020 flow diagram displaying the selection method of qualified studies. From Page et al. [44].

The result of the database searches is reported in the PRISMA 2020 flow diagram in Figure 1. The excluded records include technical reports (*n* = 31), medical device reports (*n* = 23), statistical reports (*n* = 23), not from BRICS countries (*n* = 39), does not include rehabilitation (*n* = 12), not based on PHC (*n* = 7), does not address concepts of interest, such as service protocol, practice model, and service guideline (service delivery related) (*n* = 111), and study protocols (*n* = 6).

### Characteristics of included records

This scoping review did not include any Level I research, and the majority of studies are classified as Level V (expert opinion and text and opinion) and Level III [1–3] studies. There was a total of 32 records from research conducted in BRICS countries, namely Brazil (five studies), Russia (one study), India (three studies), China (four studies), and South Africa (15 studies). There were also four global records that were text and opinion pieces, and a review that included studies from BRICS countries: Brazil (*n* = 1) and South Africa (*n* = 3). All records were published between 2011 and 2021 (Table 5).

**Table 5.** Characteristics of included records (*n* = 32).

Type of study	No. of studies	All BRICS included	Brazil	Russia	India	China	South Africa	Global
Case study	3		1		1		1	
Analytical cross-sectional	9	0	3		1	3	2	
RCT’s	2	0			1		1	
Case control	1	0					1	
Text and opinion	14	0		1		1	9	3
SR and syntheses	1							1
Interpretive and critical research	2	0	1				1	
Total	32	0	5	1	3	4	15	4

### Review findings

Using the WHO Framework for Action [45] and WHO Guide for Action [12], the findings concerning practice models, service protocols, and service guidelines and their application in integrating rehabilitation services into PHC are described in terms of governance and leadership, finance, service delivery, health workforce, and technology and information systems (Table 5). A detailed description of the review findings is presented in Table 7 (Online Supplementary File).

#### Practice models

Practice models are the conceptual frameworks and philosophies that underpin healthcare delivery. The transactional nature of practice models considers the relationship between client populations’ problems, the goals of healthcare practitioners, and the goals of healthcare systems [46]. Of the 32 included records, 27 were charted based on models for integrating rehabilitation and primary care identified by McColl et al. [9] (Table 5).

#### The clinic model

The clinic model report outpatient clinics as the most common method of integrating rehabilitation in PHC described in three records [47–49] from China, Brazil, and Russia, respectively. Due to inadequate transportation, long waiting times, and poor health awareness, clinic access is difficult in all three countries. All clinics have a doctor-led hierarchical structure, and in China and Russia, there is limited access to rehabilitation professionals [47,48]. Governance, financial, and information concerns were highlighted in Russia, including delays in regulatory framework approval and limited resource access [47] (Table 6).

**Table 6.** Summary of review findings for selected records (*n* = 32).

Concepts		Integration of rehabilitation services	Country	Health system building blocks	Challenges	No. of studies
Practice models	Clinic model (*n* = 3)	Out-patient clinics [19–21]	Russia (*n* = 1) China (*n* = 1) Brazil (*n* = 1)	Service delivery	Poor integration of rehabilitation services into communities with services offered far from home with long waiting times [21]. Lack of affordable rehabilitation services, because of poverty, inadequate transportation, and lack of health awareness [48].	27
				Leadership and governance	Delays in the regulatory framework to support the organisation and network of the clinics [19].	
				Health workforce	Inconsistency between the personnel structure and services [19]. Services do not include rehabilitation professionals, only community health workers trained by medical professionals. Lack of rehabilitation professionals [20].	
				Financing	Poor financial and economic structures to provide comprehensive medical services, including diagnostics, prevention, treatment, rehabilitation on one base [19].	
				Information systems	The backlog of the development of the information support system [19].	
	Outreach (*n* = 3)	Tele-health and mobile health as “virtual outreach” [22–24]	Brazil (*n* = 1) South Africa (*n* = 3) India (*n* = 1)	Leadership and governance	Legal clarification of the roles and responsibilities of professionals and patients. Rehabilitation professionals are under-represented. Confidentiality, the suitability for complex conditions, and legal issues are of concern [23].	
				Financing	Lack of funding to develop and support telemedicine programs [23].	
	Case management (*n* = 2)	Integrated community centres [26,27]	China (*n* = 2)	Service delivery	Patients had limited influence on the management of their case [27].	
					Facility-based appointment system affected the patients’ preferred continuity of a relationship with the same team of HCWs and free choice in whom to consult [27].	
	Shared care (*n* = 9)	Task shifting [22,28–33]	South Africa (*n* = 4) Brazil (*n* = 2) India (*n* = 1) Global (*n* = 2)	Leadership and governance financing	No accreditation and federal funding to the Family Health Support Teams [33]. Balanced care sharing model did not support multidisciplinary practice in rehabilitation services [50].	
		Task sharing [28,29]			Funding by territories or states and municipalities may result in reduced health professional involvement with a shift to the private sector [33]	
				Health workforce	Training of mental health specialists is focussed mainly on individual care with little inclusion of community based perspectives and an understanding of rural contexts [22]	
		Telehealth or online practice [22,23]		Financing	High data costs for mobile phone use for telemedicine and online practice [22]	
	Self-management (*n* = 3)	Self-help interventions [22]	South Africa (*n* = 2) Global (*n* = 1)		Besides evidence, social, political, and economic contexts greatly influence the adoption and implementation processes of models [22]	
		Support groups [29]			Applying the ICCCF to fragmented PHC services is challenging [29]	
		Psychoeducation [29]				
		Healthy lifestyle interventions [22]			None reported	
		Addressing caregiver burden [22,34]				
		Vocational interventions [22]				
	Community based rehabilitation (CBR) (*n* = 6)	Community Rehabilitation Workers (CRW) [28,51–56]	South Africa (*n* = 4) India (*n* = 1) Global (*n* = 1)	Health workforce	Poor translation of CBR theory into practice, health and other professionals’ poor understanding of CBR, and a lack of willingness to move away from individual treatment or the medical perspective towards the social and rights-based approach to managing health in communities [34].	
				Service delivery	Little or no evaluation of the clinical effectiveness or logistical or financial viability of audiology programmes was reported [24].	
		Community involvement [35]			Implementation of CBR requires obtaining community involvement and using available resources for CBR roll-out which is difficult to achieve [34].	
				Health workforce and service delivery	Lack of resources, continuous training for HCWs, and safety and security concerns for those working in communities [34].	
Service protocols	Screening protocols (*n* = 2)	Protocol for identification of problems for rehabilitation [57]	Brazil (*n* = 1) SA (*n* = 1)	Health workforce	Involvement of professionals who would use the protocol was advantageous to the acceptance in practice but as with all service protocols some resistance to change was encountered [57].	4
		Low-cost hearing screening tool [58]			Protocol addressed communication using virtual technology as well as quality of care, however, as a standalone project did not consider organisational goals which resulted in poor follow-up to local clinic services which were affected by a long waiting period [58].	
	Intervention protocols (*n* = 2)	Task shifting in mental health services [59]	South Africa (*n* = 1) China (*n* = 1)	Health workforce and service delivery	Protocol provides for some limited structured and improved communication with reservations in terms of the ability of CMHWs to deliver treatment and the availability of specialist staff to provide supervision and support [59]. Lack of occupational therapists in the study even though aspects addressed fall within their scope of practice [60].	
		Peer support service [61]		Service delivery	Although the suggested intervention and staffing protocol may address the gaps in mental health service delivery in rural resource-scarce settings in South Africa and may reduce costs, the protocol did not address quality of care or organisational goals and uncertainties exist in the implementation [59].	
				Leadership and governance	Meeting organisational goals depends on the capacity and willingness of district health management who were not part of the project [59].	
Service guidelines	Organisational policies (*n* = 4)	Guidelines for human resource requirements [62,63]	South Africa (*n* = 4)	Service delivery	None reported	9
		Private healthcare guidelines for reimbursement [64]				
		Guidelines for a mental health CBR service [65]				
	Professional standards (*n* = 3)	Professional standard guidelines for speech therapy [66]	Brazil (*n* = 1) India (*n* = 1) South Africa (*n* = 1)	Health workforce and service delivery	Structural and organisational challenges, high patient loads, and space constraints affected practice as well as referrals [36].	
		Guidelines for community rehabilitation services for children with intellectual disabilities [37]			Lack of healthcare services and visits by physiotherapists and speech therapists mean several healthcare needs of the children were not met [37].	
		Guidelines to integrate mental healthcare into chronic care [36]		Health workforce	Professional standards guidelines for nutritionists and no other rehabilitation professionals in supporting Ward-Based Outreach Teams [36].	
	Reliability of rehabilitation process (*n* = 2)	Home based rehabilitation guidelines [38]	Brazil (*n* = 1) South Africa (*n* = 1)	Service delivery	The service infrastructure for transportation/travel time for therapists to conduct home visits requires revision [38]	
		Guidelines for integrating rehabilitation services for children living with HIV [39]			Loss to follow-up after the initial assessment, because of financial constraints, lack of time, and transport restrictions [39]	

#### The outreach model

Three records [51,67,68] on telehealth and mHealth services address outreach. These services provided a virtual outreach model for integrating rehabilitation services, including psychosocial rehabilitation with PHC e-health [67], and tele-audiometry to screen hearing in South Africa [51]. In Brazil, a telehealth network connected remote and rural areas to secondary care and specialised services, including rehabilitation, at reduced costs [68]. Governance challenges include unclear health professional roles, insufficient rehabilitation services, confidentiality, and payment for services [68].

#### Case management

Case management is the third model in integrated rehabilitation service delivery, outlined in two reports. Lee [69] describes an integrated service where patients with chronic physical conditions receive rehabilitation and continuity of care in one setting case managed by their doctor. The second source addresses the difficulties of facility-based visits without case management, which leaves patients with no continuity of treatment, no interpersonal relationship with one multidisciplinary team member, and no choice in PHC service locations [70].

#### Shared care

Shared care, described in nine records is the highest evidenced model for integrating rehabilitation in PHC [49,50,52,67,68,71–74]. These records describe task-shifting and task-sharing in community-based teams, as well as telemedicine or online practice with collaboration between an interdisciplinary team of healthcare workers and others [67]. Five records emphasise task-shifting for integrating rehabilitation services, particularly in mental health services [52,67,71–73], and include rehabilitation integration into PHC involving community health workers (CHWs). Although Hanlon [71] is the first to propose this practice model, it was previously effectively applied in the Community Care for People with Schizophrenia in India experiment, providing evidence for task-shifting in mental healthcare [52].

Telehealth or online practice projects involving health professionals are another type of shared care that uses peer-to-peer sharing, learning, mentoring, and coaching [68] to promote mental healthcare in South African PHC practice. Workforce challenges related to the latter study include expensive data costs and training of mental health specialists who do not focus on community-based perspectives and understanding rural situations [67]. Moreover, in South Africa data expenses affect the feasibility of supporting telehealth and online practice services [67]. There are numerous governance challenges in terms of shared care, and in their proposed balanced care sharing model in PHC mental healthcare services, Goldberg et al. [50] fail to consider any inter- or transdisciplinary practices in rehabilitation services and focus on curative services.

#### Self-management

Three records emphasise self-management in PHC rehabilitation service integration [53,67,71]. Empowering patients, carers, and communities with information and support to self-manage has improved outcomes in the innovative care for chronic illnesses framework [71]. Similarly, PHC mental health services in South Africa show good evidence for functional outcomes and quality of life-based on self-management by families, carers, and patients [67]. Self-help programmes with support groups, vocational interventions, healthy lifestyle interventions, carer burden reduction, psychoeducation, and regular functional recovery reviews are suggested [67].

#### Community-based rehabilitation

Six records report community-based rehabilitation (CBR) as the final model for integrating rehabilitation services [51–56]. Four of the six records [51,54–56] specifically address CHWs, community rehabilitation workers (CRWs), and community involvement in CBR programmes where trained CHWs or CRWs offer rehabilitation services in the community [51,52,55,56]. Cobbing et al. [56] use a randomised control trial to evaluate mobility rehabilitation using a CBR approach for HIV-positive patients by CRWs trained and supervised by physiotherapists in South Africa. Bhutta [51] also recommends using trained CRWs in CBR to provide general or targeted audiology and speech therapy services in low-resource settings. Swanepoel [55] reports the successful screening of pre-schoolers in a low-resource area for hearing and visual loss using unemployed community members trained as CRWs.

Augustine [54] emphasises the importance of community involvement in CBR to identify and develop programmes to provide rehabilitation services in schools for intellectually impaired children in India. They found that community involvement increases school support and ownership, and that CRW rehabilitation services at school and home visits reduce stigma and care burden. However, gaining community access and facilitating community participation as well as the requisite resources for CBR are time consuming and difficult [53]. These challenges in conjunction with the lack of understanding of CBR by health and other professionals, and a reluctance to shift away from a curative medical perspective to recognise the social and rights-based approach to community health management impede CBR [49,71]. The safety of CHWs while providing community services is also emphasised [53] because it affects service delivery, as does the lack of CBR rehabilitation programme efficacy outcomes [51].

#### Description of service protocols

Service protocols are multidisciplinary and include pre-set criteria to be followed in defined situations to achieve a set of standards to support specified patient care, which motivate quality teams to improve communication and service quality within organisational goals [11]. Four records [60,75–77] describe screening or intervention service strategies to improve service quality. Both the screening protocols include rehabilitation integration in PHC. The Protocol for Identification of Issues for Rehabilitation, based on the International Classification of Functioning, Disability, and Health, was developed in Brazil in a case study. This protocol guides the screening of patients for referral and case management in an interdisciplinary team in which rehabilitation professionals, such as occupational therapists, physiotherapists, and SLTs working in family healthcare centres are included [75].

The second service protocol includes only SLT services. CRWs in South Africa were trained and implemented a low-cost preschool hearing screening routine using a mHealth point-of-care diagnostic and cloud-based data storage, surveillance, and referral system [76].

Both intervention protocols consider rehabilitation in mental health in PHC and do not mention rehabilitation professionals, such as occupational therapists. The first protocol uses task-shifting and presents a service protocol for a hypothetical interdisciplinary team needed to populate the integrated primary mental healthcare framework in South Africa. However, the protocol does include rehabilitation for adult mental healthcare users in a rural setting [77] where community mental health workers provide psychosocial CBR programmes and weekly group and individual therapy. Governance and service delivery challenges in the protocol include the lack of involvement of governmental district health management structures. Community mental health workers face limited structured communication and a lack of specialist staff to supervise and support them, which affect their service delivery and quality of care [77].

A second intervention protocol is described in a cross-sectional analytical study from China [60]. In this study, a peer support service protocol for rehabilitation services improves perceived independence in activities and the social skills of people with mental illness attending PHC community rehabilitation. The service protocol requires a high level of supervision and organisational resources, but after one year, supervision can be reduced, making the service protocol sustainable [60]. In this study, psychiatrists, community doctors, and clinical psychologists trained and supervised peer service providers online [60].

#### Description of service guidelines

Service guidelines refer to principles or criteria that guide action and are carried out to adhere to organisational policies and professional standards to ensure the best possible outcomes and reliable rehabilitation services [10]. Nine records from the review discuss service guidelines [78–86]. Of the nine records, four [78–80,87] report on service guidelines for human resource requirements, professional standards, and the reliability of the process in PHC rehabilitation in the public and private health sector in South Africa.

The four sources describe service guidelines for human resource requirements within the PHC re-engineering approach proposed by the Department of Health in South Africa. Daviaud and Subedar [87] consider human resource requirements for clinic and community services but exclude rehabilitaion professionals. Later guidelines, published in 2020, included criteria for ward-based PHC outreach teams in a community-orientated primary care approach [78]. This article includes the geographic delineation of teams supported by a dietician, physiotherapist, occupational therapist, pharmacist, social worker, and hospital-based specialists to improve district health services [78]. However, professional standards guidelines are only included for nutritionists to support ward-based outreach teams, despite the inclusion of all services at PHC clinics, community health centres, and environmental and school health [82]. However, the study supports the recommendations of Homer [80] who proposes guidelines for the development of a mental health CBR service in South Africa that includes occupational therapists as part of the team [80]. The private sector guidelines for rehabilitation services in South Africa include services offered in the community by rehabilitation professionals in private practice, such as physiotherapists, occupational therapists, and SLTs, which are mainly available to those with medical insurance [79].

Service guidelines for professional standards are the second component considered for the integration of rehabilitation into PHC and are reported by three records [81–83]. In Brazil, Molini-Avejonas et al. [81] analyses the professional standards guidelines from the Ministry of Health for SLTs in family health centres [81]. They found the SLTs working in a multidisciplinary team met all guidelines related to professional service. Other studies on professional standards in India [83] and South Africa identify challenges, such as a lack of an interdisciplinary approach and government interdepartmental collaboration. In India, the Health and Education Departments and local self-governments require linkage to the District Mental Health Programme to deliver community rehabilitation services of an adequate standard to children with intellectual disabilities [83]. A lack of inclusion of all rehabilitation professions is also a concern since visits by physiotherapists and SLTs are omitted from the interdisciplinary approach [83].

A study in South Africa to develop a district mental healthcare plan to integrate mental health services into chronic care proposes collaborative care packages offered by CHW outreach teams for depression, alcohol use disorders, and schizophrenia in alignment with the WHO’s Mental Health Gap Action Programme. Services depend on psychoeducation provided by PHC nurses and doctors and community-based psychosocial rehabilitation groups facilitated by auxiliary social workers and lay counsellors [82]. Service delivery in terms of professional standards is also impacted by structural and organisational challenges, high patient loads, and space constraints, which affect practice and referrals [82].

The reliability of the process, the third component of service guidelines for the integration of rehabilitation in PHC, are reported on by two records [84,86]. The first study completed an analysis of the time spent by the health interdisciplinary team in home care based on guidelines provided by the Brazilian Ministry of Health for the Better at Home Program [86]. This determined whether the process in relation to staffing and the organisation of the service met the needs of the population served. The second is a study to confirm guidelines for the reliability of rehabilitative care in South Africa for children living with HIV. The recommendations from this study include the change in the process of community screening and timely ­referral to decentralised rehabilitative care in the home and community [84].

## Discussion

This study set out to identify and describe the practice models, service guidelines, and protocols for the integration of rehabilitation services, including those offered by rehabilitation professionals (occupational therapy, physiotherapy, and SLTs) in PHC in BRICS countries. Overall, the majority of records included in the review are classified as low level of evidence that consists mostly of opinion pieces. The highest number of records came from South Africa, and four each came from Brazil, China, and India. Only a few records from Brazil and South Africa present research completed by rehabilitation professionals. The high number of records from South Africa in comparison to the other BRICS countries may indicate that research in South Africa is routinely published in English, followed by India.

### Practice models

Data extracted from the literature are discussed using McColl et al.’s [9] six practice models for integrating rehabilitation into PHC, namely the clinic, outreach, case management, shared care, self-management, and CBR models. Although these models are distinct, they may overlap, and more than one may be implemented simultaneously. The reviewed studies indicate that rehabilitation is not integrated into the described programmes, which typically involve only one or two professional groups with skills in a single discipline and no interdisciplinary rehabilitation services at the PHC level. However, research in Brazil emphasises the importance of interdisciplinary teams in the community and the integration of all practice models within PHC.

#### The clinic model

Outpatient clinics are the preferred model for public PHC services in both South Africa and India [88,89]. This is also the case for Russia [63] and China [57] where health reforms for integrated preventive, therapeutic, and rehabilitative services based on the International Classification of Functioning, Disability, and Health have increased the number of PHC clinics and health centres. These reforms have encouraged geographic co-location of a number of health professionals in each PHC setting, where the physical proximity has improved interactions and knowledge exchange [90]. In China, many patients are required to keep their own health records as they are referred to different facilities and health professionals, including rehabilitation services, using a centralised facility-based appointment system. However, patients reported little continuity of care and felt that their freedom to choose and remain with their healthcare team members for continuity of care was not respected [70].

Popular as the clinic model is, the rehabilitation workforce remains the greatest challenge in outpatient clinics, particularly in China and Russia, due to the low number of trained occupational therapists, physiotherapists, and SLTs [64,91]. The attempt to incorporate rehabilitation services, such as physiotherapy into PHC clinics in Russia relied on a single accountable physician trained to provide all medical and rehabilitation services in these clinics [47]. Most rehabilitation services in China continue to be physician-led and assisted by “rehabilitation therapists” who are mid-level workers trained in techniques used by occupational therapists, physiotherapists, SLTs, and traditional Chinese medicine [70]. The integration of rehabilitation services remains poor, and access to services is a challenge when the clinic model is used to provide PHC [49]. Thus, a move away from clinics to family health centres in the community in Brazil has brought integrated rehabilitation services close to patients’ homes, overcoming challenges that impact service utilisation and health outcomes [92]. De Jesus Mari [93] concurs that the only way to achieve rehabilitation integration for mental health services is moving out of the hierarchical fragmented medical model services with long waiting times at PHC clinics. This model allows the interdisciplinary team to share knowledge and take co-responsibility for cases using a matrix support tool [93].

However, challenges remain, such as services being unequally distributed across Brazil and concerns about the lack of clarity in terms of the roles of the different health professionals within the system [49]. Governance challenges, resulting in delays in the regulatory framework required to support the organisation and network of the PHC services at the clinic level, are reported as a challenge, particularly in Russia [47]. These bureaucratic inefficiencies and lack of direction to operationalise services within the PHC package negatively influence the capacity to deliver services [17] and integrate rehabilitation services in PHC.

#### The outreach model

In contrast to the clinic model, outreach services emanate from a PHC facility to people who cannot access them in their usual facility setting. The integrated community-based services offered in Brazil reflect an outreach model that includes home visits, which is not described in other countries [49,93]. Outreach has also been achieved in the form of telehealth, e-health, and mobile Health (mHealth) [51,67,68]. The main advantage of these services, reported in Brazil and South Africa, is the reduced cost of providing services. In South Africa there is high evidence for telehealth [94], which also overcomes geographical boundaries and the need for travel to provide rehabilitation in mental health and audiometry screening, promoting universal health coverage [51,67]. In Brazil, the telehealth network achieved similar goals by providing access to specialised services, including rehabilitation [68]. Many of the leadership and governance challenges in the legal clarification of the roles and responsibilities of service providers and users of telehealth reported previously [51,67,68] may have been resolved during the COVID-19 pandemic when telehealth services became a necessity. Due to data costs and connectivity in middle lower-income countries, the question arises of whether telehealth is an efficient and effective method to integrate rehabilitation services into PHC [67].

#### The shared care model

Both the clinic model and outreach model suggest the use of shared care to integrate rehabilitation services in PHC, either through task-shifting (the delegation of components of care) or through task-sharing (a collaboration with shared responsibility for care when specific tasks are assigned to different HCWs) [71]. This supports the health workforce challenges to rehabilitation service integration in PHC, where a lack of trained rehabilitation professionals remains a concern, particularly in the BRICS and other low- and middle-income countries [34]. Shared care involving trained CHWs or mid-level workers is recommended, but this form of transdisciplinary working requires comprehensive training by appropriate health professionals working in interdisciplinary contexts [95].

While a shared care model has been proposed for low-income South African communities [50], specific rehabilitation professionals, such as occupational therapists and physiotherapists are not included, particularly for mental health. In this instance, the lack of doctors in a rural setting resulted in task-shifting of all training and supervision of mental health CHWs to mental health nurses [73]. CHW training should be completed by practitioners in the relevant specific rehabilitation discipline [80], particularly when the goals of the service include establishing self-help and support groups and livelihood and income-generating opportunities, which fall within the scope of occupational therapy [71]. Furthermore, having only nurses and medical specialists train CHWs negates the transdisciplinary approach, undermines the role of rehabilitation professionals, jeopardises the contribution of specific disciplines within rehabilitation [47], and fails to recognise their unique competencies, skills, and knowledge as essential components of PHC [96]. Lack of supervision of CHWs by qualified rehabilitation professionals may reduce the effectiveness and perceived need for rehabilitation in the long term [97]. Evidence for the effectiveness of task-shifting and -sharing strategies in South African PHC for rehabilitation is lacking [67]. However, studies report effective rehabilitation services in PHC for children living with HIV [72], and the potential benefits of transdisciplinary mental health management in Brazil, including communication and recognition between healthcare workers and strengthened team bonds to provide more comprehensive, integrated care [49].

#### The self-management model

The self-management model advocates for increased empowerment of patients and the community in the ownership of their health and wellbeing and is applicable within any of the models described above. The literature emphasises these services that should prioritise the transfer of knowledge and skills to individuals, families, and the community in collaboration with mental healthcare users to promote mental health and prevent mental illness [80]. Self-management can be supported by online or telehealth services to reduce healthcare professionals’ workload while capturing a larger audience [67]; however using self-management in fragmented PHC services is challenging [71], and therefore integrated PHC services are necessary.

#### The community-based rehabilitation model

Training CHWs to provide rehabilitation services is an effective CBR model for integrating rehabilitation in PHC and includes the delivery of home-based community care. Data extracted on CBR emphasise improved functional and quality of life outcomes, the significance of continued education and training, appropriate supervision of CHWs, and the involvement of the community in achieving health goals [51,53,98]. According to Augustine [54], training community members as CHWs helps overcome community stigma and opposition to community involvement, but the safety of CHWs and the time needed for developing community involvement remain concerns [53]. A rehabilitation programme for intellectually impaired children in India, which included interdisciplinary collaboration, report that positioning the community at the core of identifying needs and developing programmes to deliver rehabilitation services enhanced CBR’s success as a model for integrating rehabilitation in PHC [54]. Success in CBR programmes in South Africa is reported and includes improved functional mobility in people with HIV where CHWs were trained in physiotherapy skills [56] and CHWs trained by SLTs to effectively screen children for hearing loss [51,55].

### Service protocols

Service protocols facilitate service delivery within multi- and interdisciplinary teams and this review found protocols for screening and intervention. These protocols should allow health professionals to apply clinical judgement to implement them [99].

The collaborative development of a screening protocol involving all team members, including occupational therapists, physiotherapists, and SLTs, was successful in guiding patient referral and management within the interdisciplinary community-based teams in Brazil. Implementation was facilitated by team involvement and increased protocol acceptability in practice [75]. Similarly, a low-cost hearing screening protocol for preschool children was developed and implemented in South Africa, which supported the transfer of SLT skills through CHW training [76]. While the protocol was successful for screening, it lacked organisational goals. This resulted in poor follow up and long waiting periods for hearing assessments and intervention at local clinics [76], impacting the integration of rehabilitation services.

A service protocol for intervention was proposed for the assessment and treatment of adult mental healthcare users by a hypothetical multidisciplinary team in a rural South African setting. The protocol involves task-shifting to CHWs for delivering psychosocial rehabilitation and interpersonal therapy groups but does not include rehabilitation professions, such as occupation therapists, which is concerning. Moreover, the absence of consideration for district health management goals resulted in uncertainties during project implementation [77]. In China, a peer support programme was approved for individuals with severe mental illness in which mental healthcare users provide support under the supervision of community doctors and clinical psychologists. However, the lack of involvement of occupation therapists and physiotherapists in training may have affected the quality of care for specific interventions related to daily living skills, social skills, and fine motor skill practice [87].

### Service guidelines

Rehabilitation service guidelines are essential in PHC to ensure equal distribution, affordability, and accessibility while adhering to organisational policy and professional standards to make the service acceptable to healthcare workers and consumers [80].

#### Organisational policies

South Africa implemented guidelines for ward-based outreach teams in a community-orientated primary care approach to improve district health services and save costs [78]. These guidelines specify the geographic delineation and composition of PHC teams, conditions of employment, and managerial oversight for supervision and accountability [78]. Furthermore, the guidelines stipulate that a hospital or district-based dietician, physiotherapist, occupational therapist, pharmacist, medical specialist, and social worker must support the team; however, the training and supervision of the CHWs within the ward-based outreach teams exclude rehabilitation services [78]. In the private healthcare sector, guidelines for rehabilitation services are limited to identifying codes for prescribed minimum benefits that cover individual health and rehabilitation services for those with medical insurance or paying out of pocket [79].

#### Professional standards

Professional standards guidelines are crucial to ensure standards of care are achieved by healthcare workers [87]; however, non-adherence to these guidelines remains a threat to the integration of rehabilitation services in PHC. Studies from Brazil [81] and India [83] show that weak guidelines affect care standards. A study from South Africa indicates that non-compliance with guidelines results in poor referral rates by PHC nurses to community healthcare workers [88]. A crucial aspect of the successful integration of rehabilitation services into PHC is the development of clear comprehensive guidelines. Nonetheless, it is equally important for the individuals responsible for implementing such guidelines to ensure their appropriate execution [35].

#### Guarantee reliability of the rehabilitation process

Bôas and Shimizu [86] analysed the time spent by the multidisciplinary health team in home care to ensure the reliability of the rehabilitation process in Brazilian services for the “Better at Home Program” domiciliary care [86]. They found differences within the interdisciplinary PHC team for procedures in the home, with physiotherapists following the community home visit guidelines for intervention and assessment of patient safety, resulting in sustained improvements in patients’ compliance [86]. Similarly, Maddocks et al. [84] suggest that service guidelines for integrated rehabilitation for children living with HIV should consider the context in which they live since home programmes provided by hospital-based rehabilitation professionals are found to be unsustainable due to the limited availability of trained professionals, financial constraints, lack of time, and transport restrictions. They also suggest that the guidelines should include community screening and simple home-based treatment strategies by CHWs to improve the reliability of the rehabilitative process [84].

### Limitations

As only data published or translated to English were included in this review, unknown valuable data from studies published in other languages may exist.

## Conclusion

The integration of rehabilitation services in BRICS countries is impeded by a shortage of rehabilitation professionals and limited access to services, especially in remote areas. To address this challenge, the involvement of CHWs and CRWs in transdisciplinary teams has been proposed. Recruiting and training unemployed community members as CRWs can improve access to rehabilitation services, especially in areas where healthcare professionals are scarce. Peer support workers have been identified as a potential model for improving rehabilitation outcomes and integrating rehabilitation in PHC through shared care and self-management. The CBR model is confirmed as the most effective approach to integrate rehabilitation in PHC, emphasising community involvement in service provision through trained CHWs and CRWs. Implementing CBR programmes can improve rehabilitation outcomes, ensure effective service delivery, and promote community-based inclusive development. Therefore, in low- and middle-income countries with limited access to rehabilitation services, the use of CHWs, CRWs, peer support workers, shared care, self-management, and the CBR model present promising approaches to integrate rehabilitation in PHC.

## Supplementary Material

Supplemental Material

## Data Availability

The data that support the findings of this study are available from the corresponding author, Lebogang Maseko, upon reasonable request.
