# Supplementary material for: Service guidelines, models, and protocols for integrating rehabilitation services in primary healthcare in Brazil, Russia, India, China, and South Africa: a scoping review
Source: Disabil Rehabil. 2023 Dec 9;46(22):5144–57. doi: 10.1080/09638288.2023.2290210 (PMC11552699; doi:10.1080/09638288.2023.2290210)
Supplement: Supplemental Material [file IDRE_A_2290210_SM1363.docx]

**Table 7 (online supplementary file): Detailed description of review findings from records according to service protocols, guidelines and models (n=32).**

| **PRACTICE MODELS** | | | | | | | | | |
| --- | --- | --- | --- | --- | --- | --- | --- | --- | --- |
|  | **Reference** | **Country** | **Aim** | **Methods/Type of study** | **Service providers/ Setting/Context** | **Participants under study** | **Services described** | **Classification: Model, protocol or guideline** | **Key findings from source** |
| 1 | Vialkov, A.I., Skvirskaia, G.P., Son, I.M., Senenko, A.S., Kupeieva, I.A., Rosanov, V.B., Leonov, S.A., Bilalov, F.K., et al., 2017. The actual approaches to reformation of medical organizations providing out-patient care to population. *Problemy Sotsial'noi Gigieny, Zdravookhraneniia i Istorii Meditsiny*, *25*(4), pp.216-220. | Russia | Development of organizational functional model of primary health care in actual conditions with the purpose of supporting accessibility and quality of care in out-patient conditions. | Text and opinion Study | PHC - only a single physician responsible for all care  Doctors should replace physiotherapists | None | Primary medical care and rehabilitation in the Russian Federation | Clinic model | Introduction of one stop PHC for the provision of PHC with a single responsible physician. Clinics will have specialist trained GP to organize:   - general medical departments - specialized advisory assistance, - medical prophylaxis, diagnostics, - medical rehabilitation, - medical and social care - day hospital. Outpatient care PHC medical units consist of independently functioning units offering various services.   Doctors in medical units should gradually replace physiotherapists and physical therapy physicians. |
| 2 | Chen, S., Lei, Y., Dai, H., Wu, J., Yang, Z., & Liao, X. (2020). Community-based rehabilitation service in Chengdu, Southwest China: a cross-sectional general survey. BMC health services research, 20(1), 1-7.*planning and management*, *35*(1), e196-e209. | China | Evaluate the capacity of rehabilitation service in primary health centres in Chengdu, China (vertical integration). | Cross sectional analytic study | Rehabilitation physicians and traditional Chinese medicine (TCM) physiotherapists primary health institutions (THCs) | 379 primary health centres responded to this survey | Evaluated:   - rehabilitation practitioners - physical therapy equipment - space: no. of plinths and no. of therapeutic rehabilitation rooms. | Clinic model | Chinese government has financed PHC with marked increase in services  Rehabilitation therapy available in:   - 88.9% of all primary health centres - 92.2% of community health centres (CHCs) in cities - 87.5% of township health centres (THCs) in the rural area. |
| 3 | de Sousa, F. S. P., Jorge, M. S. B., Vasconcelos, M. G. F., Mont'alverne de Barros, M. M., Quinderé, P. H. D., & Gondim, L. G. F. (2011). Building the mental health care network with the matrix support tool. *Physis*, *Revista de Saúde Coletiva* *21*(4), 1579. | Brazil | In-depth discussion about reality based on Brazilian psychiatric reform | Interpretive and critical research | nurses, psychologists, doctors, social workers, occupational therapists, pharmacists; community health workers | 47 higher education professionals and mental health service providers | Psychiatric outpatient clinic | Clinic model  Shared care | Important tool for reorganizing the flow of mental health care in a network  Being a priority in producing comprehensive, continuous and quality care for people in psychological distress  Allows construction of a therapeutic follow-up not limited to the borders of a given service, but diluted in several instances, articulated by a reference team. |
| 4 | Mapanga, W., Casteleijn, D., Ramiah, C., Odendaal, W., Metu, Z., Robertson, L., & Goudge, J. (2019). Strategies to strengthen the provision of mental health care at the primary care setting: an evidence map. *PloS one*, *14*(9), e0222162. | Global  BRICS county included | Determine evidence for strategies to strengthen the provision of mental health care at the primary care setting | Systematic review and Syntheses | Different cadres of health workers offering PHC services-(highly qualified mental health workers to health workers with shorter training and fewer qualifications) | 306 full text articles | General primary care, specialist psychiatric care or any psychiatric treatment offered to those with a mental illness  Measures of function, quality of life, improvement in symptoms, as well as patients’ acceptability of the interventions implemented within PHC across high, middle or low-income country settings | Outreach model  Shared care | Strategies that empower families, carers and patients as well as integration of care/collaborative interventions, and e-health interventions. However, most of the evidence came from high-income countries, such as USA, UK and Australia,  Need to understand that besides evidence, social, political and economic contexts greatly influence adoption and implementation processes. |
| 5 | Fatehi, F., Taylor, M., Caffery, L. J., & Smith, A. C. (2019). Telemedicine for Clinical Management of Adults in Remote and Rural Areas., *New Imaging Technologies and Digital Health*, 439-461. | Global - BRICS county included | Overview of telemedicine services | Text and opinion- | All providers of clinical services from specialist secondary care to PHC units- case study in Brazil | None | Connection of specialist secondary care primary care units, located in remote and rural areas to in 88.0% of Minas Gerais state | Outreach model (‘virtual outreach’)  Shared care model | Reasons for success: Support of public managers   - Service provision though a collaborative network, - Government‐academia partnership, - Ease of use of the systems, - Diversity of telehealth activities, - Economic viability |
| 6 | Bhutta, M. F. (2019). Models of service delivery for ear and hearing care in remote or resource-constrained environments. *The Journal of Laryngology & Otology*, *133*(1), 39-48. | Global - BRICS county included | Overview of service delivery for ear and hearing care in remote or resource-constrained environments | Text and opinion- | Speech and language therapists | None | Hearing care | Outreach model | Tele-audiometry has been utilised in a number of applications, including remote diagnostic audiometry and otoacoustic emissions testing, |
| 7 | Lee E. One-stop boost for chronically ill patients Public consultation on primary care reform (2010) ProQuest [Internet]. *South China Morning Post;* Hong Kong | China | Implementation of the primary care reform | Text and opinion- | medical consultations, dietician physiotherapy nursing care | none | New integrated community health centres (polyclinics) that place all primary care services under one roof | Case management model | Patients with chronic conditions have access to medical professionals as well as rehabilitation in one setting.  Doctor will act as case manager for patients. |
| 8 | Liu, C., Wu, Y., & Chi, X. (2017). Relationship preferences and experience of primary care patients in continuity of care: a case study in Beijing, China. *BMC Health Services Research*, 17(1), 1-11. | China | Exploring the association between patient relationship preferences and their correlations with patient experience in continuity of care. | Cross sectional analytic study | doctors, nurses and pharmacists with 11% other including rehabilitation staff | 700 patients attending PHC centres and clinic | one centre and five outreach stations with PHC community health team | Case management model | Patients prefer high level of freedom of choice and sustained individual relationship with HCWs.  Strong patient preference for free choice is not aligned with strong continuing relationship with primary care. Recommends: increased attention to informational and managerial continuity as they are more likely than relational continuity to impose a direct impact on patient care outcomes |
| 9 | Diniz, L. M., Rhodes, G. A. C., Abreu, M. H. G., Borges-Oliveira, A. C., & Chalub, L. L. H. (2020). Home visits in primary care: Differences among professional categories and health macro-regions. Indian Journal of Dental Research, 31(3), 494. | Brazil | Review of changes in the PHC care model in Brazil | Cross sectional analytic study | Multidisciplinary community based PHC team including OT, PT, SLT, doctors, nurses, CHWs | none | Unified Health System (Sistema Único de Saúde or SUS) and Family Health teams (Núcleos de Apoio a Saúde da Família or NASFs) | Shared care model | The establishment of Family Health teams for multidisciplinary health care in the community in Brazil supported the pillars of the PHC model and health promotion. Changes in 2017 in the PHC care model threaten the teams’ multidisciplinary, weaken the community territorial approach and the principles of universality, integrity, and equity in the SUS |
| 10 | Goldberg D, Thornicroft G and van Ginneken N (2016) The role of primary care in low- and middle-income countries. In: Thornicroft G, Deb T, and Henderson C (eds) *Community Mental Health Care Worldwide: Current Status and Further Developments*. Blackwell Publishing Ltd, pp. 276–286. | Global includes BRICS country | planning and provision of mental health systems, - the need to develop in more detail models of community mental health services for low- and middle-income countries | Text and opinion | Specialist staff training, primary care staff and others in families, faith-based social services, NGOs, vocational services, peer-support services, and self-help services. | none | Community based mental health care -case finding and assessment, and psychosocial treatments | Shared care model | Balanced care model:  in low-income countries or sites, mental health provision should be invested in staff for primary health care and community settings.  Specialist mental health care staff provide training and supervision of primary care staff. Outpatient/ambulatory clinics, community mental health teams, long-term community-based residential care, and options for work and occupation. |
| 11 | Hanlon C (2017) Next steps for meeting the needs of people with severe mental illness in low- and middle-income countries. *Epidemiology and Psychiatric Sciences* 26(4). Cambridge University Press: 348–354. | South Africa | Case studies from non-governmental organisations - holistic approaches to rehabilitation, recovery and empowerment of people with severe mental illness (SMI) in PHC | Text and opinion | facility-based health workers provide outreach  CMHWs  service users and caregivers | None | The Programme for Improving Mental health care (PRIME) - specialist mental health care is mostly accessible to the community.  Primary care to provide a recovery-oriented psychosocial group intervention with task shifting and holistic approaches to rehabilitation, recovery and empowerment of people with SMI | Shared care model | Engagement with traditional and faith healers is recommended as a part of community provision of care for people with SMI, but this is rarely put into practice and even more rarely evaluated. Specialist-led community mental health care for people with SMI, with complex needs has only been tested and evaluated in South Africa mostly supported by NGOs. |
| 12 | Maddocks, S., Nakooda, M., Cobbing, S., Hanass-Hancock, J. & Chetty, V. (2021) Perceptions of care and rehabilitation for children living with HIV in KwaZulu-Natal province, South Africa. *Vulnerable Children and Youth Studies*, 16(2). 151–165. | South Africa | To explore the perceptions and insights of HCWs and stakeholders, on the care of children living with HIV (CLHIV) in a semi-rural area to inform and improve the rehabilitation framework | Text and opinion- qualitative study | Healthcare workers, community care workers, educators, social workers, non- government organisation employees, and spiritual and traditional leaders | doctor  physiotherapists  occupational therapist  social workers  community care workers  HIV counsellor  educators  nurse  dietitian | Care or education to CLHIV | Shared care model | The biopsychosocial viewpoint of disability experienced by CLHIV was adopted. Barriers to care - stigma and denial of HIV infection by carers, poor multi-disciplinary team functioning. Ongoing education of healthcare workers, educators and carers as well as and task-shifting of care to lay community members for disability screening and referral to improve rehabilitative care for CLHIV |
| 13 | Vergunst R (2018) From global-to-local: rural mental health in South Africa. *Global Health Action* 11(1): 1413916 | South Africa | Current situation regarding rural mental health services- approaches to make it context appropriate using task shifting or sharing, and formal vs informal health care systems | Case control study form | General nurses community service workers.  mental health nurses’ | none | Mental health nurses’ - prescribe medication in rural areas in only 38.7% of 160 facilities for population of more than 17 million.  Community-based approaches, including self-help and peer-support.  No mental illness rehabilitation service delivery platform exists in many areas in South Africa | Shared care model | South African Mental Health Care Act difficult to implement in rural areas. Rural areas in South Africa are particularly under-serviced in psycho-social rehabilitation programmes, - task shifting can close mental health service gaps where culture shapes illness experience with a relationship between informal (faith and traditional healers) and formal (clinicians) |
| 14 | Blose, S.B., Doeraj, S., Padia, S., Pillay, K., Reddy, K. & Chetty, V. (2021) Healthcare professionals’ perceptions of community-based rehabilitation in KwaZulu-Natal, South Africa. *African Journal of Primary Health Care &amp; Family Medicine*  13(1). AOSIS Publishing: 1–9 | South Africa | To explore perceptions of healthcare professionals on CBR across four public healthcare facilities in KZN | Text and opinion descriptive study | Health care professionals  PT. OT, SLT, audiologists, dieticians, pharmacists, dental therapists, psychologists, social workers, nurses and doctor | Physiotherapists  occupational therapist  speech and language therapists, audiologists, dieticians, pharmacists, dental therapists, psychologists, social workers, nurses and doctors | Hospitals linked to the University of KwaZulu-Natal community- based primary healthcare training platform - two district hospitals, two hospitals with district regional and tertiary services | Self-management model  CBR model | Themes ‘*the community-based rehabilitation conundrum’* refers to challenges in healthcare professionals’ understanding of the strategy of CBR  ‘*Community-based rehabilitation enablers’* refers to facilitators for effective CBR- community healthcare workers (CHWs) were key role players for the successful implementation of CBR. Barriers to the implementation of CBR- lack of resources, poor translation of CBR theory into practice, lack of continuous training and poor safety and security. |
| 15 | Augustine A (2016) Community-based rehabilitation for children with intellectual disability: Experiences from endosulfan affected areas in India. *Disability, CBR and Inclusive Development* 27(3): 132–140. | India | Describes a Community-Based Participatory Approach (CBPA) for children with intellectual disability | Text and opinion – case studies | BUDS schools and BUDS Rehabilitation Centres | Families of the children, rehabilitation workers  community members | Day care, life skills training, academic training and nutritious food to children with disabilities  Income generation activities and creation of employment opportunities | CBR model | Local ownership and participation of the community ensured cultural uniqueness. Special neighbourhood groups (NHG) met to support the activities of the centre. The issues of limited resources and sustainability were resolved by identifying and incorporating the existing assets in the society, an effort spearheaded by LSG. |
| 16 | Chatterjee, S., Naik, S., John, S., Dabholkar, H., Balaji, M., Koschorke, M., Varghese, M., Thara, R., et al. (2014) Effectiveness of a community-based intervention for people with schizophrenia and their caregivers in India (COPSI): A randomised controlled trial. The Lancet 383(9926) | India | Community care for People with Schizophrenia in India (COPSI) trial to compare a collaborative community-based care intervention with standard facility-based care | Parallel-group, randomised controlled trial | Community health workers with 10 years of schooling and good interpersonal skills supervised by supervised by psychiatric social workers and treating psychiatrists | 187 participants to collaborative community-based care plus facility-based care  95 to the facility-based care alone 253 (90%) participants completed follow-up to month 12 | Individualised rehabilitation strategies to improve the personal, social, and work functioning of participants  dealing with stigma and discrimination  Link to self-help groups and other methods of user-led support  Networking with community agencies - help with social inclusion, legal benefits, and employment opportunities | Shared care model | The collaborative community-based care plus facility-based care intervention is modestly more effective than facility-based care, - reducing disability and symptoms of psychosis. in settings where services are scarce e.g. in rural areas |
| 17 | Cobbing S, Hanass-Hancock J and Myezwa H (2017) A Home-Based Rehabilitation Intervention for Adults Living With HIV: A Randomized Controlled Trial. *Journal of the Association of Nurses in AIDS Care* 28(1). Elsevier Inc: 105–117 | South Africa | aimed to assess the impact of a 16-week disability-inclusive home based rehabilitation (HBR) program on the quality of life, perceived disability, functional capacity, and mobility of adult PLWHIV | Randomized controlled trial | Four community health care workers trained and supervised by physiotherapists | 34 participants with HIV in each of an intervention and control group on ART for more than 6 months, mobility limitations, according WHODAS 2.0 questions S1 and S7 | Home based rehabilitation intervention. According to specific physical impairments. | CBR model | The intervention was implemented by CHWs who lived in the same communities suggesting that a task-shifting approach was a feasible and potentially cost-saving in providing rehabilitation for PLWHIV.  Other disciplines should contribute to the training and supervision of community health workers to provide access to a wider range of rehabilitation services  Home Based Rehabilitation can address the functional deficits, is safe and likely to improve QoL. |
| 18 | Swanepoel DW (2019) We Used Smartphones to Screen Young Children for Vision and Hearing Loss [analysis]. Available at: https://0-search-proquest-com.innopac.wits.ac.za/docview/2305420484/fulltext/308A4C5526EF47F6PQ/1?accountid=15083 | South Africa | develop, implement and evaluate vision and hearing care models in poor communities | Text and opinion | Unemployed community members and trained as community health workers | 8023 children screened across 271 preschools | To provide vision and hearing screening at preschool centres using smartphones with pre-installed mobile health technology.  Second hearing screening included otoscopy, by the project audiologist,  referral to public health diagnostic audiology and vision services | CBR model | Effective task shifting  Test time was just over 2 minutes, cutting typical test times in half  More than a hundred children in this sample were diagnosed with a hearing or visual impairment and are now receiving treatment |
| **SERVICE PROTOCOLS** | | | | | | | | | |
|  | **Reference** | **Country** | **Aim** | **Methods** | **Service providers/ Setting/Context** | **Participants under study** | **Services described** | **Classification: Model, protocol or guideline** | **Key findings from source** |
| 19 | Souza, M. A., Ferreira, F. R., César, C. C., Furtado, S. R., Coster, W. J., Mancini, M. C.and Sampaio, R. F. (2016). Development of a first-contact protocol to guide assessment of adult patients in rehabilitation services networks. *Brazilian Journal of Physical Therapy*, *20*, 148-157. | Brazil | Development of a protocol l to standardize collection of functional information based on the ICF | Case study -protocol development | physical therapists, occupational therapists,  speech pathologists, nutritionists, psychologists,  social workers, pharmacists, and physical educators | 61 rehabilitation professionals and rehabilitation managers from the public rehabilitation services of Belo Horizonte, as well as rehabilitation researchers from Universidade Federal de Minas Gerais | improving communication among multidisciplinary team and levels of service guiding the patient’s pathway throughout the rehabilitation network |  | The protocol improves quality of service and interterm communication by standardising the first contact between the user and the rehabilitation service. The active participation of professionals in the construction of the protocol meant it is in line with the reality of rehabilitation services and increased professional compliance Behavioural changes in clinical practice still require adaptation |
| 20 | Yousuf Hussein, S., Swanepoel, D. W., Mahomed, F and Biagio de Jager, L. (2018). Community-based hearing screening for young children using an mHealth service-delivery model. *Global Health Action*, *11*(1), 1467077. | South Africa | Protocol for smartphone-based hearing screening program for preschool children operated by CHWs in community- based ECD centres. | Cross sectional analytic study | speech and language therapists  community health workers | 6424 children (3–6 years) in 250 ECD centres in Mamelodi East and West | smartphone-based hearing screening  Mapping ECD centres in community  Results to parents and teachers - text messages |  | The hearScreen^TM^ Smartphone-based hearing screening can be used by CHWs to detect unidentified hearing loss in young children. The test operator test quality increased during the project. An overall referral rate for hearing loss was 24.9% but only 39.4% attending follow-up at a local clinic, of whom. |
| 21 | Petersen, I., Lund, C., Bhana, A., Flisher, A. J., & Mental Health and Poverty Research Programme Consortium. (2012). A task shifting approach to primary mental health care for adults in South Africa: human resource requirements and costs for rural settings. Health policy and planning, 27(1), 42-51. | South Africa | Determines a hypothetical human resource mix protocol framework for district adult mental health services using task shifting with dedicated low cost CMHWs at the community and clinic levels | Text and opinion | clinical psychologist psychiatric nurses  primary health care nurse  mental health counsellor  community mental health workers | None | psychosocial rehabilitation groups for people with severe mental disorders  individual counselling for maternal depression and group counselling for depression and PTSD |  | Task shifting may substantially reduce the expected number of health care providers otherwise needed to close mental health service gaps at PHC level.  Task shifting in real world settings runs the risk of CMHWs being tasked with the provision of treatment packages without adequate training, supervision and support |
| 22 | Fan, Y., Ma, N., Ma, L., Xu, W., Steven Lamberti, J., & Caine, E. D. (2018). A community-based peer support service for persons with severe mental illness in China. *BMC psychiatry*, *18*(1), 1-10. | China | Evaluation of a protocol for the implementation of a peer support t services for persons with severe mental illness (SMI) | Cross sectional analytic study | community doctors, social workers, clinical psychologists, psychiatrists | 12 peer - mental health patients with personal and social performance scale score (PSP) of more than 50.  50 MHCUs with SMI in community rehabilitation or health care centres. | peer activities including daily life skills, social interpersonal skills, knowledge, entertainment, fine motor skills and exercise, self-esteem and self-confidence, healthy life style, and emotional support. |  | .79.2% of MHCUs with SMI were satisfied with the peer support 70.8% wanted to continue. 41.7% reported better social communication skills and 33% of peer service providers found their own mood improved  Peer support service for patients with SMI can be sustainably implemented within communities but peer service providers need increased support at the beginning of the programme |
| **SERVICE GUIDELINES** | | | | | | | | | |
|  | **Reference** | **Country** | **Aim** | **Methods** | **Service providers/ Setting/Context** | **Participants under study** | **Services described** | **Classification: Model, protocol or guideline** | **Key findings from source** |
| 23 | Council for Medical Schemes (2019) Draft: Primary Health Care Services. Available at: https://www.medicalschemes.com/files/PMB Review/DraftPHC.pdf | South Africa | Guidelines for a omprehensive PHC services with emphasis on Alignment of the PMB package | Text and opinion | Occupational Therapy  Physiotherapy | none | The committee recommends:   - Rehabilitation services - Assistive Devices and consumables - Mobility devices - Positioning devices - Prosthetics, orthotics and orthopaedic shoes - Daily living devices - Vision devices - Hearing devices   Rehabilitation - measures that assist individuals, who experience or are likely to experience disability, to achieve and maintain optimum functioning in interaction with their environments | Service guidelines | Specification of a comprehensive set of services. Assistive devices and technologies’ primary purpose is to maintain or improve an individual’s functioning and independence to facilitate participation and enhance overall well-being. The following devices are proposed;  Rehabilitation interventions that include training, exercises, and compensatory strategies, education, support and counselling for  • prevention of the loss of function • slowing the rate of loss of function  • improvement or restoration of function  • compensation for lost function, maintenance of current function. |
| 24 | Daviaud E and Subedar H (2012) Staffing Norms for Primary Health Care in the context of PHC Re-engineering Report to the National Department of Health. Available at: https://www.samrc.ac.za/sites/default/files/files/2016-07-14/StaffingNorms.pdf4/StaffingNorms.pdf | South Africa | Guidelines to inform short- and medium-term planning, deployment and training requirements for staffing norms in each component of the PHC services | Text and opinion | Doctor  Specialised Psychiatric Nurse  Professional nurse  Enrolled nurse Nursing Assistant  Pharmacist  Councillor Optometrist  Psychologist  Dental Therapist  Oral Hygienist  Optometrist  Nutritionist  Assistant Health Promoters  Community Health Worker (CHW)  Home-Based Carers (HBC) | none | Service for  ANC  Genetic Services,  Complex Conditions,  Chronic Conditions  Mental Health:  For Outreach  School Health  Clinic services  CHC services  Specialised Team | Service guidelines | additional staff requirements for the introduction of new services, outreach teams as well as district specialist teams, increased school health services and includes all HCWs including CHWs and home-based carers but no rehabilitation staff  the target number of staff is based on utilisation and does not include the staff required to keep facilities open after-hours  introduction/input of psychology mid-level worker  Rehabilitation services-: Physiotherapy, Occupational Therapy, Speech/Hearing Services not defined |
| 25 | Homer S (2014) Improving Health and Access to Health Services through Community-Based Rehabilitation Chp 9. In: *Occupational Therapy in Psychiatry and Mental Health*. Oxford: John Wiley & Sons, Ltd, pp. 126–147 | South Africa | Occupational therapy in mental health in community-based rehabilitation  Guidelines-  ● community service programmes  ● health promotion and prevention  ● components of a CBR service | Text and opinion | Occupational therapists  Community rehabilitation workers | none | mapping  participatory rural appraisal  needs of the community consumer needs  mobilising the community in CBR  ● education about mental health and disability  ● information about how to access local health resources  ● the development of healthy lifestyles for clients and the broader community  ● early detection of people with mental disabilities  ● training in activities of daily living  ● training in handling difficult behaviour  ● access to finance | Service guidelines | Studies in rural areas of South Africa and India show that for caregivers the greatest burdens are financial, disruption of daily activities/routines and disruption in family relationships Mental health problems are to be found in the community and not in hospital, it is important to understand life in that community and the issues around life events  Service providers need to develop and follow national policies for mental health, use technology appropriate for the primary care level and provide cost-effective services.  In some communities, it is essential for the occupational therapist to contact local leaders to gain permission to work in the community and have some guarantee of safety |
| 26 | Mash R, Goliath C, Mahomed H, et al. (2020) A framework for implementation of community-orientated primary care in the Metro Health Services, Cape Town, South Africa. *African Journal of Primary Health Care and Family Medicine* 12(1).1–5 | South Africa | Guidelines for the implementation of ward-based community-orientated primary care (COPC) outreach teams with community health workers. In the Western Cape | Framework development | PHC team members, - NPO or facility  10–15 CHWs professional nurse,  clinical nurse practitioner  medical officer | task team  stakeholders | 10 inter-related elements: geographic delineation of PHC teams,   - composition of PHC teams - facility-based and community-based teamwork, - partnership of government and non-government organisations - scope of practice, - information system, - community engagement - stakeholder engagement - training and development of PHC teams, - system preparation - change management | Service guidelines | A strategy is needed to clinical, support and managerial staff within the DOH are aware of and share a similar understanding of the new COPC  inform communities about the new COPC - elicit their support for the PHC teams working in their communities.  scope of practice, learning outcomes and curriculum for the pre-service training of the PHC team, and especially the CHWs, needs to be co-ordinated at the provincial level and with educational institutions. New CHWs should have a matric and acquire formal National Qualification Framework (NQF) credits with career pathways |
| 27 | Molini-Avejonas, D.R., Aboboreira, M.S., Couto, M.I.V. & Samelli, A.G.. (2014) Insertion and performance of Speech-Language Pathology and Audiology in Family Health Support Centers. *CoDAS* 26(2): 148–154 | Brazil | to evaluate implementation of guidelines for speech language therapists in Centers for Supporting the Family Health (NASF) | Cross sectional analytic study | Speech and Language therapists  doctors, nurses, nursing assistants or nursing technicians, community health workers oral health team  OTs PTs | 40 speech language therapists | Centers for Supporting the Family Health (NASF)Analysed guidelines for:   - work infrastructure, - NASF SLTs, - services provided - satisfaction with work | Service guidelines | 40% of speech language therapists consider that the family health services do not meet the proposed guidelines in NASF  reported an average of 8.9 professionals and 1.6 speech language therapists per team.  work for 40 hours per week.  services include- promotion and health prevention parent-based therapy, support to health community workers, referrals, home visits, intersectoral practice and administrative tasks. the majority were “Somewhat satisfied” with work infrastructure and referrals, and “Very satisfied” with home visits and support for health community workers with no significant difference from guidelines |
| 28 | Nujum, Z.T., Anilkumar, T. V., Vijayakumar, K., Anish, T.S. & Moosan, H. (2012) A Framework for healthcare provision to children with intellectual disability. *Asia Pacific Disability Rehabilitation Journal* 23(4): 54–66. | India | needs assessment for development of guidelines for CBR service delivery for children in the BUDS schools | Cross-sectional survey was | teachers  limited visits from physiotherapists/ speech and language therapists | medical records, interviews with the parents, and clinical assessment of 202 children at 11 registered ‘Buds’ schools in Kerala | Intervention guidelines required for:   - nutritionist - occupational therapy - physiotherapy - speech and language therapy | Service guidelines | guidelines for the Medical Colleges and Health Services to deliver services to children using a multidisciplinary approach, needs to be developed  Interventions needed included self-help and social skills training, sensory motor and cognitive stimulation and parent counselling with parent training in rehabilitation home programmes |
| 29 | Petersen, I., Fairall, L., Bhana, A., Kathree, T., Selohilwe, O., Brooke-Sumner, C., Faris, G., Breuer, E., et al. (2016) Integrating mental health into chronic care in South Africa: The development of a district mental healthcare plan. *British Journal of Psychiatry* 208(s56): s29–s39 | South Africa | to develop district mental healthcare plan (MHCP) guidelines to integrate mental healthcare into chronic care | Text and opinion -descriptive analysis | PRIME collaborative care package, community-based programme  By nurses,  lay counsellors  auxiliary social workers from the DoSS, Mental Health Society and NGOs and CHWs | Participatory theory of change (ToC) - key stakeholders including service managers, service providers and patients  Qualitative interviews with 79 individual interviews with stakeholders and 4 focus groups. | Comprehensive care packages for depression, alcohol use disorders and schizophrenia enable integration at the organisational, facility and community levels, supported by a human resource mix | Service guidelines | In terms of counselling and psychosocial rehabilitation  ●lay counsellors, - marginalised status, unclear roles; low confidence; and poor suitability  ●poor follow-up of patients referred to them for counselling.  ●a high default rate and poor tracing of individuals who defaulted  Developed implementation guidelines for district and facility managers, role clarification of the different team members - occupational therapy only considered necessary at a tertiary level. |
| 30 | Bôas MLDCV and Shimizu HE (2015) Time spent by the multidisciplinary team in home care: Subsidy for the sizing of staff. *ACTA Paulista de Enfermagem* 28(1): 32–40. | Brazil | to analyse time spent by multidisciplinary team in home care.to develop guidelines | Analytical cross sectional descriptive study | nursing staff  physiotherapists  dieticians  physicians | 214 patients in 245 home visits while observing 441 procedures. | Home Care Program (HCP) of Distrito Federal.  Physiotherapy -evaluation/adaptation of the residential environment  Physiotherapy session (respiratory, motor, neurological and/or trauma)  Guidance to the caregiver and/or patient | Service guidelines | time spent in direct care by  nursing staff, of 30.2 hours  physiotherapists with 11.9h,  dieticians with 9.4h  physicians with 8.9h.  Indirect care from 65.3 hours to 20.3h was represented travelling and guidance to the caregiver, family and/or patient  The domiciliary physiotherapy was for chronic diseases, with the main goal being motor rehabilitation for stroke, osteoarthritis and fractures.  Travel time was 41.5% of the total period which impacted negatively on the productivity  Time spent will support guidelines for staff numbers, infrastructure and planning and organization of the service |
| 31 | Maddocks ST, Mthethwa L and Chetty V (2020) Child functioning and disability in children living with human immunodeficiency virus in a semi-rural healthcare setting in South Africa. *African Journal of Primary Health Care & Family Medicine* 12(1): 1–8. | South Africa | to understand the level of functioning and access to rehabilitative care of children living with HIV (CLHIV) to inform integrated rehabilitative guidelines. | Cross sectional analytic survey | rehabilitation professionals  psychologist  dentist  HIV counsellor | 45 caregivers of children receiving treatment for HIV | Assessed using Washington Group/United Nations International Children’s Emergency Fund (UNICEF) Module on Child Functioning  Referred to rehabilitation professionals  most accessed care for an initial assessment  17 caregivers did not return for follow-up treatment due to distance and cost of travel |  | Need for guidelines for routine, disability-screening practices for CLHIV as part of their standard HIV care using CBR programmes as well as mental health service inclusive of all health professionals based on a task shifting model should be considered. |
